# Cannabis Addiction and the Brain: a Review

**DOI:** 10.1007/s11481-018-9782-9

**Published:** 2018-03-19

**Authors:** Amna Zehra, Jamie Burns, Christopher Kure Liu, Peter Manza, Corinde E. Wiers, Nora D. Volkow, Gene-Jack Wang

**Affiliations:** 1 Laboratory of Neuroimaging, National Institute on Alcohol Abuse and Alcoholism, National Institutes of Health, 10 Center Drive 31, Room B2L124, Bethesda, MD 20892 USA; 2 National Institute on Drug Abuse, National Institutes of Health, Bethesda, MD 20892 USA

**Keywords:** Substance use disorders, Dopamine, Marijuana, THC

## Abstract

Cannabis is the most commonly used substance of abuse in the United States after alcohol and tobacco. With a recent increase in the rates of cannabis use disorder (CUD) and a decrease in the perceived risk of cannabis use, it is imperative to assess the addictive potential of cannabis. Here we evaluate cannabis use through the neurobiological model of addiction proposed by Koob and Volkow. The model proposes that repeated substance abuse drives neurobiological changes in the brain that can be separated into three distinct stages, each of which perpetuates the cycle of addiction. Here we review previous research on the acute and long-term effects of cannabis use on the brain and behavior, and find that the three-stage framework of addiction applies to CUD in a manner similar to other drugs of abuse, albeit with some slight differences. These findings highlight the urgent need to conduct research that elucidates specific neurobiological changes associated with CUD in humans.

## Introduction

Cannabis is the most commonly used substance of abuse in the United States after alcohol and tobacco (Carliner et al. 2017). In the US, cannabis use increased from 4% to 9.5% between 2001 and 2002 and 2012–2013 and the prevalence of Cannabis Use Disorder (CUD) increased from 1.5% to 2.9% in the same time (Hasin et al. 2015). Despite these increases in cannabis use and CUD, attitudes towards cannabis use have softened: adult and adolescent perceptions of cannabis use risk have decreased since 2001 (Hasin et al. 2015; Carliner et al. 2017). These shifting attitudes have intergenerational consequences as offspring of parents who are early-onset cannabis users and who meet criteria for CUD are more likely to become early-onset cannabis users themselves (Henry and Augustyn 2017). With increases in cannabis use and decreases in perceived risk, it is necessary to reevaluate the addictive potential of cannabis (Carliner et al. 2017; Hasin 2018).

**Fig. 1:**
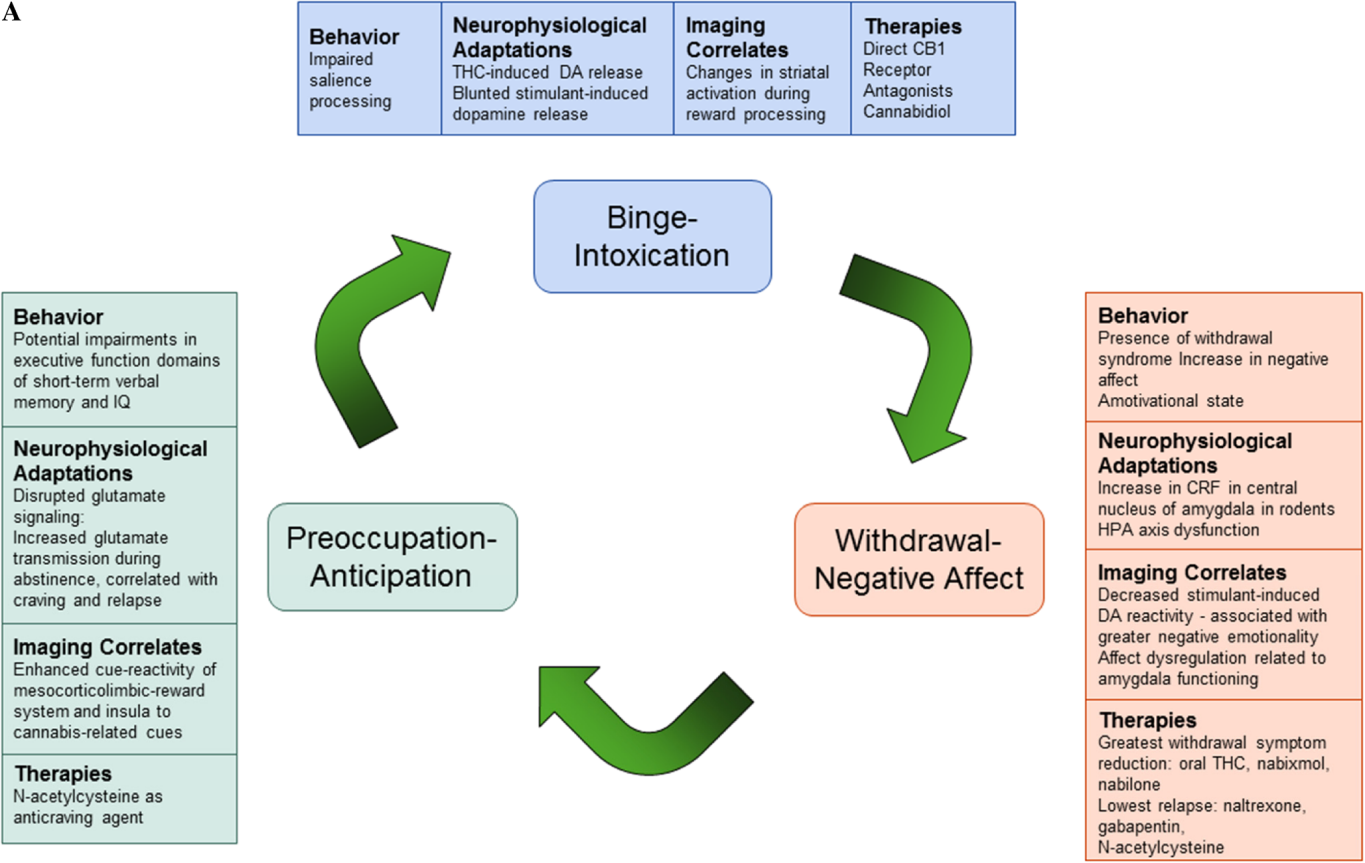

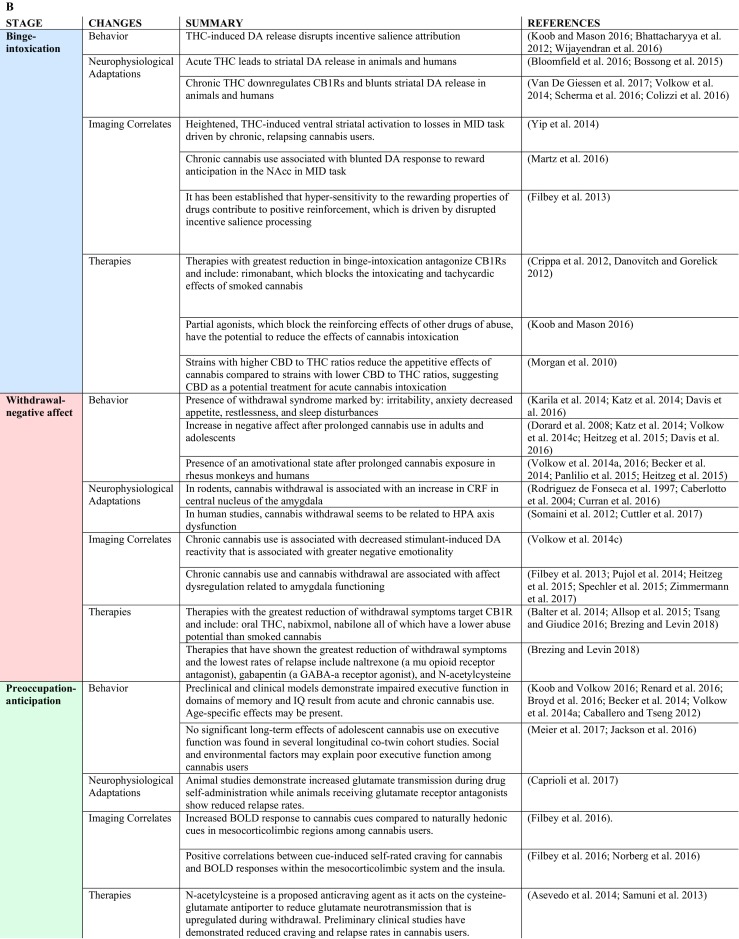
**a**. Model of neurocircuitry and correlating disruptions in brain function and neurophysiology that contribute to behaviors underlying drug addiction. **b**. Summary of the changes in neurocircuitry associated with each stage

In this review, we explore the nature of cannabis addiction through a prominent model of drug addiction (Koob and Volkow 2016). We first explain the model, which proposes a dysregulation of motivational circuits in three stages of addiction: binge/intoxication, withdrawal/negative affect, and preoccupation/anticipation. Second, we summarize empirical evidence for preclinical and human studies on the acute and long-term effects of cannabis use on the brain and behavior (similar to those of other drugs of abuse). Third, we review potential therapeutic agents for CUD that may provide further evidence for dysregulation in motivational circuits in CUD. After reviewing the acute and chronic effects of cannabis use on the brain and behavior and treatment options for cannabis abusers, we discuss whether there is empirical evidence that the three stages of addiction apply to CUD (Fig. 1 provides an overview of the current literature supporting this model).

## Theoretical Model of Addiction

Koob and Volkow (2016) define drug addiction as a “chronically relapsing disorder” marked by compulsive drug seeking and intake, loss of control in limiting intake, and the emergence of a negative emotional state when access to a drug is prevented. This model proposes three stages of addiction with disturbances in three major neurocircuits: the binge/intoxication stage driven by changes in the basal ganglia; the withdrawal/negative affect stage driven by changes in the extended amygdala; and the preoccupation/anticipation driven by changes in the prefrontal cortex (PFC). Within these domains, Koob and Volkow (2016) describe neuroadaptations in 18 subsystems including the ascending mesocorticolimbic dopamine system, corticotropin-releasing factor (CRF) in the central nucleus of the amygdala, and corticostriatal glutamate projections.

The binge-intoxication stage of addiction is characterized by an excessive impulsivity and compulsivity to use drugs despite negative consequences associated with such use. This stage involves hyperactivation of the mesocorticolimbic dopaminergic reward pathway of the brain associated with the positive reinforcement of the rewarding effects of drugs. A hallmark of the binge/intoxication stage is an impairment in incentive salience, whereby drug-associated cues and contexts associated with the initial exposure to a drug are attributed exaggeratedly high rewarding properties and become conditioned to elicit dopamine (DA) release. This incentive salience dysfunction appears to drive DA signaling to maintain motivation to take the drug upon exposure to conditioned-cues and even when its pharmacological effects lessen, secondary to the development of tolerance (Koob and Volkow 2016).

The withdrawal/negative affect stage is then triggered by opponent-process responses following binge episodes. These opponent-process responses are marked by within-systems and between-systems neurobiological changes that drive the loss of motivation towards non-drug rewards and impaired emotion regulation seen in this stage. Within-systems neuroadaptations include changes in the function of brain reward systems including decreased dopaminergic signaling in the nucleus accumbens (NAcc) and dorsal striatum that result in an elevation of reward thresholds for non-drug reinforcers, which contributes to amotivation. Between-systems neuroadaptations include dysfunction of neurochemical systems that are not primarily involved in the rewarding effects of drugs of abuse; this includes changes in brain systems involved in stress responses such as increased CRF release in the amygdala and HPA-axis dysfunction. The changes resulting from opponent-processes responses drive characteristic symptoms of a withdrawal symptom such as increased anxiety-like responses, chronic irritability, malaise, and dysphoria during acute and protracted abstinence from a drug of abuse (Koob and Volkow 2016).

The preoccupation/ anticipation stage is implicated in the reinstatement of substance use following abstinence. Executive control over craving and impulsivity is key in maintaining abstinence and is mediated by the PFC. The preoccupation/anticipation stage is marked by dysregulation of signaling between the PFC and areas of the brain that control decision making, self-regulation, inhibitory control and working memory and might involve disrupted GABAergic and glutamatergic activity (Koob and Volkow 2016). Behaviorally, this translates into excessive salience attribution to drug-paired cues, decreases in responsiveness to non-drug cues and reinforcers, and decreases in the ability to inhibit maladaptive behavior (Koob and Volkow 2016).

## Evidence

### Acute Effects and Insight into Reinforcing/Addictive Properties of Cannabis

All drugs of abuse increase DA release — a key neurobiological process that generates their reinforcing effects (Koob and Volkow 2016). Here we evaluate the acute changes in DA circuitry associated with cannabis intake in preclinical and clinical studies that provide basis for the reinforcing effects of cannabis. While the two main constituents of cannabis are delta9-tetrahydracannabinol (THC) and cannabidiol (CBD), THC seems to be responsible for cannabis’ addictive potential due to its psychoactive properties and associated effects on brain dopaminergic function. Acute THC administration elicits striatal DA release in animals (Ng Cheong Ton et al. 1988) and humans (Stokes et al. 2010; Bossong et al. 2015; Bloomfield et al. 2016). However, another study found no evidence for THC-induced DA release (Barkus et al. 2011); this may be because THC induces quantitatively less DA release than psychostimulants such as methylphenidate or amphetamine (Volkow et al. 1999a). Nonetheless, these findings suggest that THC increases DA release similar to other drugs of abuse.

Several animal models of cannabis exposure have been established in rodents and non-human primates (Panlilio et al. 2015). In studies with rodents, neurophysiological methods such as intracranial microinjection, microdialysis, and single-unit electrophysiological recording techniques are used to study the acute effects of THC and other cannabinoids in the brain directly (Oleson and Cheer 2012; Panlilio et al. 2015). Behavioral methods include the use of place conditioning, drug discrimination, intracranial self-stimulation, or intravenous self-administration to study the reinforcing effects of cannabinoids in vivo (for further details see: Maldonado and Rodriguez de Fonseca 2002; Tanda and Goldberg 2003; Maldonado et al. 2011; Panlilio et al. 2015; Zanda and Fattore 2018). Robust intravenous self-administration paradigms in animals have been difficult to establish. That is, in rodents THC is unable to sustain intravenous self-administration (Lefever et al. 2014), whereas squirrel monkeys have found to self-administer THC; suggesting differences in species. However, other behavioral methodologies, such as drug discrimination and conditioned place preference paradigms, reveal the rewarding effect of THC and other cannabinoids (Maldonado and Rodriguez de Fonseca 2002; Tanda and Goldberg 2003; Maldonado et al. 2011; Oleson and Cheer 2012; Panlilio et al. 2015).

In rodents, THC-induced DA release is associated with increased intracranial self-stimulation in key reward pathways of the brain (Katsidoni et al. 2013). Likewise, low doses of a cannabinoid-1 receptor (CB1R) agonist in the PFC increased spontaneous firing and bursting rates of ventral tegmental area (VTA) DA neurons, which was associated with potentiated salience of fear memories in rats (Draycott et al. 2014). THC elicits striatal DA release by activating CB1R, which are co-localized with DA receptors in the striatum and substantia nigra, regions implicated in salience processing (Wijayendran et al. 2016). This suggests that the endocannabinoid system (eCS) is involved in regulating DA release during salience attribution (Bloomfield et al. 2016), and that acute THC dysregulates the dopaminergic and endocannabinoid systems which then leads to impairments in salience processing (Wijayendran et al. 2016). These preclinical findings may provide a biological basis for human studies which show impaired salience processing after THC administration. In one study, THC-potent cannabis was found to increase attentional bias towards cannabis-related stimuli in cannabis users during a computer-based dot-probe behavioral task (Morgan et al. 2010). In a separate fMRI task, healthy participants performed a visual oddball paradigm; THC administration resulted in making non-salient stimuli appear more salient (Bhattacharyya et al. 2012). Together, these pre-clinical and clinical findings reveal that THC administration has reinforcing properties that alter salience processing via increased dopaminergic signaling like other drugs of abuse (Morgan et al. 2010; Bhattacharyya et al. 2012; Draycott et al. 2014; Wijayendran et al. 2016; Bloomfield et al. 2016).

### Long-Term Effects of Cannabis: Behavior and Cognition

Chronic cannabis use is associated with an increased risk of developing substance use disorders (SUD); about 9% of those who use cannabis present with characteristic symptoms of dependence according to DSM-IV criteria (Volkow et al. 2014a). Diagnoses of cannabis abuse and dependence in the DSM-IV did not include withdrawal due to uncertainty of its diagnostic features (Katz et al. 2014) In the DSM-5, however, cannabis abuse and dependence fall under a diagnosis of CUD which now includes withdrawal from cannabis. Withdrawal was added as a diagnostic criteria for CUD as it is often accompanied by increased functional impairment of normal daily activities similar to those seen in other SUD (Karila et al. 2014; Katz et al. 2014; Davis et al. 2016). Symptoms of cannabis withdrawal also seem to appear in a similar time course and manner as withdrawal from other substances (Karila et al. 2014).

A clinical diagnosis of cannabis withdrawal includes irritability, anger or aggression, nervousness or anxiety, sleep difficulty, decreased appetite or weight loss, restlessness, depressed mood, and physical symptoms causing significant discomfort such as shakiness or tremors, sweating, fever, chills, and headaches (Karila et al. 2014; Katz et al. 2014). Typically, symptoms of cannabis withdrawal occur 1 to 2 days after cessation of heavy use and can last between 7 and 14 days (Davis et al. 2016). The most common symptoms observed during cannabis withdrawal include irritability, anxiety, decreased appetite, restlessness, and sleep disturbances (Oleson and Cheer 2012; Panlilio et al. 2015; Curran et al. 2016; Gates et al. 2016). Sleep disturbances seem to be characterized by trouble falling asleep, decrease in total sleep time, and the presence of nightmares and strange dreams (Gates et al. 2016). The severity of withdrawal symptoms was associated with greater negative impact on normal, daily activities (Davis et al. 2016) suggesting that the effects of cannabis withdrawal seem to parallel withdrawal in other drugs of abuse.

Koob and Volkow (2016) posit that the withdrawal stage of addiction is marked by an increase in negative affect which also seems to be the case for cannabis addiction (Volkow et al. 2014c). In addition to acute withdrawal-related emotional disturbances such as irritability and anxiety (Katz et al. 2014; Davis et al. 2016), prolonged cannabis use is associated with long-term affect dysregulation. In a longitudinal study of adolescents, cannabis users consistently reported greater negative emotionality than healthy controls between the ages of 13 and 23; moreover, as healthy controls showed a decrease in negative emotionality with age, negative emotionality remained elevated for cannabis users during over the same time (Heitzeg et al. 2015). Another study of adolescents found that half of a group of adolescents undergoing treatment for cannabis withdrawal had at least one comorbid diagnosis of anxiety or depression; additionally, for these adolescents greater cannabis use was associated with increased depressive and anxiety-like symptoms (Dorard et al. 2008).

These changes in the affective state after prolonged cannabis use may also influence motivation. In both rhesus monkeys and humans, withdrawal from cannabis seems to involve the presence of an amotivational state (Karila et al. 2014; Panlilio et al. 2015; Volkow et al. 2014a, b, c, 2016). The amotivational state has been previously described as a “reduced motivation and capacity for usual activities required for everyday life, a loss of energy and drive to work and personality deterioration” (Karila et al. 2014). The origin of this amotivational state is still unknown and may be related to changes in executive function (Karila et al. 2014) and to reduced dopamine signaling after chronic cannabis use (Bloomfield et al. 2014; Volkow et al. 2014c). In rhesus monkeys, chronic cannabis smoke exposure was associated with lower motivation scores in a place conditioning paradigm, although these effects disappeared two to three months after cessation of the cannabis treatment (Paule et al. 1992). In one study of neurocognition, chronic cannabis users demonstrated impairments in verbal memory, spatial working memory, spatial planning, and motivated decision-making compared to healthy controls (Becker et al. 2014). These findings suggest that the amotivational state during withdrawal may be related to cognitive dysfunction and to reduced dopamine signaling after chronic cannabis use.

Cognitive dysfunction, specifically impairments in executive domains, after chronic cannabis use is a key feature of the neurobiological model of addiction (Koob and Volkow 2016). Deficits in executive function after chronic cannabis use have been shown in both preclinical and clinical studies. In one preclinical study, chronically administering a synthetic cannabinoid agonist to adolescent rats impaired short-term working memory in adulthood (Renard et al. 2016). Specifically, this chronic cannabinoid exposure altered PFC structure and impaired cortical synaptic plasticity from reduced long-term potentiation (LTP) in the hippocampus-PFC circuit. These findings support the theory that adolescent cannabis use causes lasting deficits in memory. However, they are likely age-specific effects as preclinical and clinical studies have demonstrated a lack of long-lasting cognitive impairments from adult chronic cannabis use (Renard et al. 2016).

Many clinical studies have investigated the long-term effects of chronic cannabis use on markers of executive function such as IQ, verbal learning, and memory. The results are varied and equivocal, as longitudinal studies with controlled confounds are difficult to establish. Volkow et al. (2014a, b, c) report that cannabis use during adolescence and young adulthood is associated with impaired functional connectivity in the brain and corresponding declines in IQ. A 2016 systematic review of 105 papers assessing the acute and chronic effects of cannabis on human cognition found that memory has been the most consistently impaired cognitive measure (both after acute and chronic cannabis use), with the strongest effects in the verbal domain (Broyd et al. 2016). The evidence for impairments in other domains of executive function such as reasoning, problem solving, and planning was less conclusive, as numerous studies found no significant differences in case-control comparisons. However, studies examining heavy users as well as early-onset users reported impaired executive function, especially when the sample was predominantly older participants (Becker et al. 2014; Broyd et al. 2016). This may suggest a conditional effect, unique to adolescent and heavy cannabis users while moderate and adult users are less vulnerable to the harmful effects of cannabis on cognition.

Despite earlier findings of impaired executive functioning in adolescent- and early- onset users, it is important to note that several recent studies found no significant long-term effects of adolescent cannabis use on executive function. Meier et al. (2018) report a longitudinal co-twin control study that showed no significant association between adolescent cannabis use and neuropsychological decline, and instead suggest social and environmental factors as explanations for poor executive function among cannabis users. This study was particularly insightful because of a large sample size (*n* = 1989) and IQ assessments prior to the onset of cannabis use (IQ obtained at age 5, 12, and 18). It demonstrated that adolescents who used cannabis had a lower childhood IQ and a lower IQ at 18 than non-users, but that there was no decline in IQ from pre- to post-cannabis use (Meier et al. 2018). These results are in line with another co-twin longitudinal study that investigated two large cohorts of twins and found no significant difference in IQ change over time between twins discordant for cannabis use (Jackson et al. 2016). However, lower baseline IQ was associated with adolescent cannabis use suggesting that social and environmental factors influence an adolescent’s subsequent cannabis use (Jackson et al. 2016). Together, these studies suggest that lower IQ may be a risk factor for cannabis abuse rather than the use of cannabis resulting in neuropsychological decline. However findings on the effects of cannabis exposure during adolescents are controversial and require investigation with prospective designs that take advantage of brain imaging technologies. The ABCD study, a prospective study that aims to follow 10,000 children as they transition into adulthood with a detailed phenotypic characterization including periodic brain imaging, would help clarify what effects cannabis consumption might have on brain development, neurocognitive function and mental illness (Volkow et al. 2017b).

### Long-Term Effects of Cannabis: Neurophysiological Changes

The chronic relapsing nature of addiction seems to involve underlying neurophysiological changes in reward, stress, and executive function circuits (Koob and Volkow 2016). Here we summarize findings about the effects of chronic cannabis use on these circuits.

Chronic cannabis abuse is modeled in animals with repeated treatments of cannabis (through smoke exposure) or THC and other cannabinoids (typically intravenous injections). Neurophysiological changes after these different methods of chronic cannabis treatment are then typically measured through electrophysiological recordings and microdialysis (Maldonado and Rodriguez de Fonseca 2002; Tanda and Goldberg 2003; Maldonado et al. 2011; Oleson and Cheer 2012; Panlilio et al. 2015).

In rats, early-life exposure to THC blunts dopaminergic response to naturally rewarding stimuli that elicit DA release later in life (Bloomfield et al. 2016). Likewise in rats, adolescent exposure to THC resulted in increased self-administration of and blunted striatal DA response to CB1R agonists in adulthood (Scherma et al. 2016). Changes in reward-related circuitry after chronic cannabis use may be related to changes in the eCS after prolonged cannabis use. The eCS has been implicated in reward-processing and reward-seeking behavior given that CB1 receptors are densely expressed in areas associated with reward processing and conditioning including the amygdala, cingulate cortex, PFC, ventral pallidum, caudate putamen, NAcc, VTA, and lateral hypothalamus (Parsons and Hurd 2015; Volkow et al. 2017a). In animals, activation of CB1 receptors seems to influence the hedonic effects of natural rewards after THC administration, suggesting that cannabis can affect reward sensitivity via activation of CB1 receptors (Parsons and Hurd 2015).

Chronic THC exposure has further been shown to downregulate CB1Rs, providing a neurobiological basis for the development of tolerance and desensitization to the rewarding effects of THC (Colizzi et al. 2016). In rodents, chronic administration of THC or CB1R agonists leads to tolerance in most responses as well as a decrease in CB1R availability in many brain areas (Maldonado and Rodriguez de Fonseca 2002; Tanda and Goldberg 2003; Maldonado et al. 2011). In cannabis users, withdrawal symptoms have also been associated with reductions in CB1R availability as assessed by [^11^C]OMAR PET imaging (Curran et al. 2016; D’Souza et al. 2016). Hirvonen et al. (2012) found that cannabis use downregulates CB1R in cortical regions, potentially altering the brain’s reward system. However, they also found that after 4 weeks of abstinence, CB1R density returned to normal in cannabis users in all regions except the hippocampus. This suggests that some neurobiological changes of chronic cannabis use are reversible (Hirvonen et al. 2012).

Chronic cannabis use and administration is also associated with neurophysiological changes in stress responsivity. In rodents, the neurophysiological changes associated with cannabis withdrawal are modeled through precipitated withdrawal through the use of rimonabant (a selective CB1R blocker) after repeated cannabinoid treatment (Maldonado et al. 2011; Oleson and Cheer 2012; Panlilio et al. 2015). Cannabinoid withdrawal in rodents is associated with an increase in the stress peptide CRF in the central nucleus of the amygdala (Rodriguez de Fonseca et al. 1997; Maldonado et al. 2011; Panlilio et al. 2015; Curran et al. 2016), which suggests the presence of between-systems changes in brain stress systems, as described by the Koob and Volkow model (2016). In addition, the eCS seems to be involved in regulating the stress response through its action on the amygdala and HPA axis (Dow-Edwards and Silva 2017; Volkow et al. 2017a). The eCS modulates interactions between the PFC, amygdala, and hippocampus which are all involved in emotional memory, anxiety-related behaviors, and drug cue-induced craving in SUD (Jasinska et al. 2014). Additionally, endocannabinoids seem to be required for feedback to normal stress responses: glucocorticoids increase the endogenous cannabinoids anandamide (AEA) and 2-acylglycerol (2-AG) in the paraventricular nucleus while CB1R antagonists increase HPA axis output. In rodents, exogenous cannabinoids seem to create a dysregulation of stress responsivity and anxiety-related behaviors (Dow-Edwards and Silva 2017).

Moreover, chronic cannabis abuse is associated with the dysregulation of stress responsivity in humans (Curran et al. 2016). Studies in cannabis users show that chronic cannabis use is related to both blunted and hyperactive stress responses (Somaini et al. 2012; Cuttler et al. 2017). Cuttler et al. (2017) found that healthy controls had an increase in cortisol levels under a stress-provoking condition compared to baseline but did not find the same increase in active cannabis users. In another study, both active and abstinent cannabis users had persistent hyperactivity of the HPA axis (measured by blood cortisol and ACTH levels) compared to healthy controls (Somaini et al. 2012). This pattern of HPA axis dysregulation is also seen in alcohol users: chronic alcohol use seems to attenuate the cortisol response to acute psychological stimulation of the HPA axis, but is related to elevated cortisol levels during alcohol intoxication and abstinence in dependent users (Stephens and Wand 2012).

In addition to its role in HPA axis dysfunction and reward processing, the hyperactivation of the eCS may also play a role in the executive dysfunction sometimes observed in cannabis use. The eCS is highly active in adolescent brain development, particularly in the PFC, a region that exercises executive function (Dow-Edwards and Silva 2017). Exogenous cannabinoids hyperactivate CB1 receptors which are expressed in pyramidal neurons and GABAergic interneurons, indicative of the regulatory role of the eCS in GABA and glutamate neurotransmission (Caballero and Tseng 2012; Volkow et al. 2017a). Activation of presynaptic CB1 receptors inhibits glutamate transmission onto GABAergic cells in the PFC, reducing the function of inhibitory prefrontal circuits. Therefore, hyperactivation by exogenous cannabinoids during development could disrupt the maturation of GABAergic interneurons in the PFC and desynchronize PFC circuits (Caballero and Tseng 2012). Thus, adolescent cannabis use may affect brain development and result in enduring alterations in the GABA/glutamate balance in the PFC (Renard et al. 2016).

Neuroadaptations in glutamatergic signaling resulting from repeated cannabis use are likely also implicated in periods of cannabis abstinence and craving (Samuni et al. 2013). This theory is supported by a review of animal studies that demonstrated increased glutamate signaling during drug self-administration and relapse, offering a potential neurochemical target for treatment in preventing craving and subsequent relapse. For example, rodent and nonhuman primate models receiving periodic injections of glutamate receptor antagonists have shown a reduction in relapse rates (Caprioli et al. 2017). Nonetheless, these findings need to be corroborated in rodents since there is conflicting evidence for whether self-administration in rodent models provides robust evidence of THC as a behavioral reinforcer (Tanda and Goldberg 2003; Maldonado et al. 2011; Panlilio et al. 2015; Melis et al. 2017).

### Long-Term Effects of Cannabis on the Brain: Neuroimaging Studies

Addiction is a recurring cycle that worsens over time and involves neuroplastic changes in the brain reward, stress, and executive function systems (Koob and Volkow 2016). Previous neuroimaging studies reveal the long-term effects of chronic cannabis use on several different brain systems including the reward, endocannabinoid, and stress systems as well as brain areas involved in emotion processing and decision making.

**Fig. 2:**
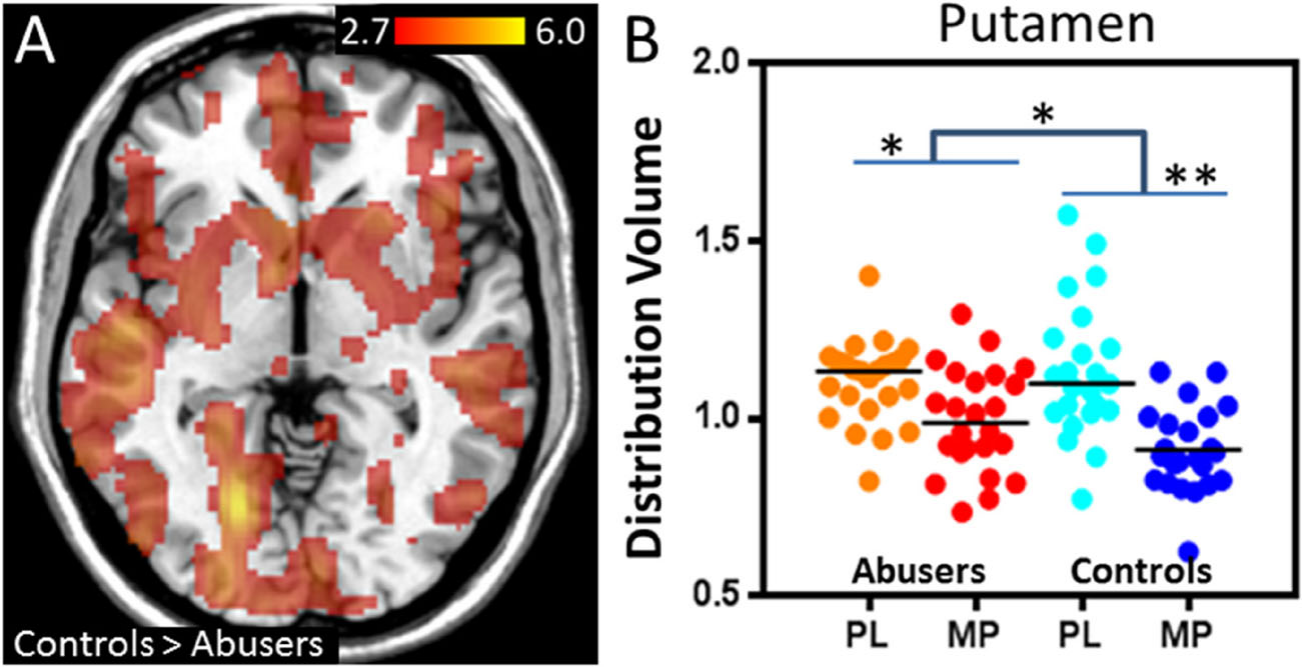
**a**. Statistical group differences in the effect of methylphenidate on the distribution volume between controls and marijuana abusers. Methylphenidate-induced decreases in distribution volumes were stronger in controls than in marijuana abusers (*p* < 0.005). There were no regions where marijuana abusers showed greater decreases than controls. **b**. Individual distribution volume values in putamen after placebo (PL) and after methylphenidate (MP) for marijuana abusers and controls. *p < 0.05, **p < 0.005. (Figure adapted with permission from Volkow et al. 2014a, b, c)

Similar to animal models of chronic THC exposure, chronic cannabis use has been shown to blunt DA response to DA-releasing stimulant drugs in the striatum with both [^11^C]-(+)-PHNO and [^11^C]raclopride PET imaging (Volkow et al. 2014c; Bloomfield et al. 2016; van de Giessen et al. 2017) and to decrease DA synthesis as assess with PET imaging with [^18^F]DOPA (Bloomfield et al. 2014) (Fig. 2). This pattern of decreased stimulant-induced DA release is also seen with chronic use of other drugs of abuse such as alcohol, cocaine, and nicotine (Koob and Volkow 2016). However, cannabis users do not show lower baseline D2/D3 receptor availability in the striatum compared to healthy controls – a pattern seen in chronic alcohol, nicotine, cocaine, opiate and methamphetamine users (Volkow et al. 1996b, 2001, 2002, 2014b, 2017c; Wang et al. 1997; Martinez et al. 2012; Tomasi et al. 2015b; Wiers et al. 2016a, 2017; Ashok et al. 2017). Moreover, the stimulant challenge led to significantly lower self-reported ratings of feeling high (Volkow et al. 2014c), and decreased brain glucose metabolism in the striatum, thalamus, and midbrain (Wiers et al. 2016b) in cannabis users versus controls. Cannabis users had higher negative emotionality and lower positive emotionality personality scores than controls, and negative emotionality scores were inversely correlated with methylphenidate-induced dopamine increases in the ventral striatum (Volkow et al. 2014c; Wiers et al. 2016b). These findings offer an explanation for decreased dopamine reactivity in the striatum during abstinence that may contribute to negative emotionality, which is consistent with lower reward sensitivity in cannabis users during the withdrawal phase of addiction (Volkow et al. 2014c). In another study, a stimulant challenge also led to blunted brain glucose metabolism in striatal regions, which was associated with craving (Wiers et al. 2016b). Together these findings from stimulant challenges indicate functional changes in the dopaminergic reward system in chronic cannabis users.

Furthermore, fMRI studies have also revealed functional and structural changes in brain areas involved in reward processing after chronic cannabis use. In one study, participants in a cannabis-dependent group had greater activation in the ventral striatum in response to losses during a monetary incentive delay (MID) task compared to healthy controls (Yip et al. 2014). Compared to controls, the cannabis-dependent participants also had smaller putamen volumes, a brain region involved in habit formation. These differences seemed to be driven by participants who were unable to stay abstinent from cannabis and were comparable to findings in tobacco smokers suggesting similar changes in reward functioning in both tobacco and alcohol addiction (Yip et al. 2014). In another fMRI study with the MID task, cannabis users in withdrawal had greater activation in the ventral striatum in response to positive incentives compared to healthy controls during the MID task, similar to findings in alcohol users (Filbey et al. 2013). Persistent cannabis use also seems to be related to a blunted response to reward anticipation in the NAcc during the MID task: in this study, even after controlling for prior and current use of other drugs, greater cannabis use was related to decreased activation in the NAcc during reward anticipation at baseline, 2 year, and 4 year follow ups (Martz et al. 2016). Together, these findings suggest that chronic cannabis use produces functional alterations in areas involved in reward processing.

A recent fMRI study investigated whether cannabis use sensitizes and disrupts the mesocorticolimbic reward processes during a hedonic cue-reactivity task. A cohort of chronic cannabis users (requiring 72 h of abstinence) showed greater BOLD response for cannabis cues compared to natural reward cues (fruit) in the orbitofrontal cortex (OFC), striatum, anterior cingulate gyrus, and VTA, regions along the mesocorticolimbic-reward pathway (Filbey et al. 2016). In cannabis users, there were also significant positive correlations between cue-induced self-rated craving for cannabis and BOLD responses within the mesocorticolimbic system and in the insula. The latter data supports the addictive model of cannabis as insula activation may serve as a biomarker to help predict relapse (Filbey et al. 2016). This brain region contributes to interoceptive awareness of negative emotional states and is differentially activated during craving (Koob and Volkow 2016). This is also consistent with prior findings that the dopaminergic reward system is reactivated during acute craving episodes (Volkow et al. 1999b, 2005; Koob and Volkow 2016). Moreover, in cannabis abusers, but not in controls, acute THC intoxication elicited activation of brain reward regions as assessed by increases in brain glucose metabolism in striatum and orbitofrontal cortex (Volkow et al. 1996a). Overall, these studies demonstrates that chronic cannabis use sensitizes the mesocorticolimbic-reward system to cannabis cues and to THC (Volkow et al. 1996a; Filbey et al. 2016). These findings suggest that chronic cannabis use affects key brain circuits involved in the reward system similar to other drugs of abuse.

In addition to changes in reward processing, chronic cannabis use also seems to affect emotion processing. Several MRI studies reveal functional and structural differences in the amygdala – a key brain structure in processing emotions – after chronic cannabis use. Compared to healthy controls, adolescents who used cannabis had lower activation in the amygdala in an emotional arousal word task during fMRI (Heitzeg et al. 2015). However, in another fMRI study, adolescent cannabis users showed greater amygdala activation to angry faces compared to controls (Spechler et al. 2015). Another study of facial emotion recognition found that during abstinence, cannabis-dependent patients performed significantly worse than controls in the identification of negative emotions suggesting a lasting impact on emotion recognition after chronic cannabis use (Bayrakçi et al. 2015). Together, these fMRI findings indicate that chronic cannabis use alters amygdala function.

The association between amygdala structure and cannabis use is relatively unclear. Some studies have found morphological and volumetric differences in the amygdala between healthy controls and cannabis users in both adolescent and adult cohorts (Gilman et al. 2014; Lorenzetti et al. 2015). On the other hand, other studies that controlled for alcohol and tobacco use found no differences in amygdala volume or shape between cannabis users and healthy controls (Weiland et al. 2015; Manza et al. 2018). A longitudinal study with cannabis users and healthy controls found no volumetric differences in gray matter at baseline or a three-year follow up (Koenders et al. 2016). Despite these inconclusive structural MRI findings, there is evidence that chronic cannabis use may contribute to emotional dysregulation through functional changes in the amygdala (Heitzeg et al. 2015; Spechler et al. 2015).

Further evidence of emotion dysregulation after chronic cannabis use is seen in fMRI functional connectivity studies with cannabis users (Pujol et al. 2014; Zimmermann et al. 2018). In one study, cannabis users showed increased resting-state functional connectivity between posterior cingulate cortex (PCC) and other regions of the default mode network (including angular gyri, medial and lateral PFC, ACC and temporal cortex), and an anticorrelation between PCC activation and insula activation. These connectivity patterns were associated with a reduction in anxiety scores suggesting an alteration of affect state that is related to changes in brain function during cannabis addiction. As the insula is involved in integrating interoceptive information for emotion, these findings suggest that cannabis may enhance visceral sensations via insula activation to modify affect state (Pujol et al. 2014). Additionally, these resting-state functional connectivity patterns lasted one month after cessation of cannabis use suggesting long-lasting changes in brain function after chronic cannabis use (although functional connectivity patterns in other networks normalized with abstinence, see Pujol et al. 2014). In another fMRI study, cannabis-dependent subjects completed task and resting state fMRI 28 days after abstinence (Zimmermann et al. 2018). During the task, in which participants were passively exposed to pictures of negative and neutral valence, negative emotional stimuli elicited larger increases in medial orbitofrontal cortex (mOFC) activity in cannabis-dependent users than in healthy controls; researchers also found greater functional connectivity between the mOFC and dorsal striatal region as well as the mOFC and amygdala compared to healthy controls during the task. Given that the mOFC is a region implicated in emotional regulation, these connectivity findings suggest the existence of persistent emotional processing alterations in cannabis-dependent users even after cessation of cannabis use (Zimmermann et al. 2018).

In addition to contributing to emotion dysregulation, cessation of chronic cannabis use is associated with the development of craving (Davis et al. 2016). Cue-reactivity is a neurobiological metric to evaluate cue-induced craving, a strong predictor of relapse for substance use (Budney et al. 2008; Wilson and Sayette 2015). A 2016 meta-analysis of cue-reactivity in regular cannabis users reported moderate to extreme cue-reactivity despite self-reports of low craving (Norberg et al. 2016). These results may indicate that cannabis users underestimate their own excessive salience, suggesting that self-reports may not accurately reflect cannabis craving intensity. Thus, excessive salience attribution to cannabis-related cues appears to be a hallmark of cannabis addiction. These studies further demonstrate the importance of collecting objective measures of craving when studying the effects of chronic cannabis use.

Finally, one of the most consistent neuroimaging findings in addiction is that of dysregulation of frontal cortical regions involved with executive function including the dorsolateral prefrontal cortex, the ACC and the inferior frontal cortex. Imaging studies investigating brain glucose metabolism, which serves as a marker of brain function, reported decreased frontal metabolism in cannabis abusers when compared with controls (Sevy et al. 2008; Wiers et al. 2016b) and in polysubstance users who consumed cannabis (Moreno-Lopez et al. 2012).

## Treatment Options

Treatments for CUD seem to target aspects of the binge-intoxication, withdrawal-negative affect, and preoccupation-anticipation stages described by Koob and Volkow (2016).

Pharmacological treatments for the binge-intoxication stage of cannabis addiction have focused on cannabinoid receptors. One mechanism of action involves direct antagonism of CB1Rs. CB1R selective antagonists such as rimonabant have been shown to block the subjective intoxicating and tachycardic effects of smoked cannabis (Crippa et al. 2012; Danovitch and Gorelick 2012). Despite the potential acute benefits, direct antagonism with rimonabant is associated with anxiety and depression (Taylor 2009; Danovitch and Gorelick 2012). Up to 10% of patients experienced anxiety and depression following use of rimonabant (Food and Drug Administration 2007). Another downfall of this therapy is that in order to avoid precipitated withdrawal, participants are required to abstain from drug use prior to administration of antagonist medications, leading to poor compliance rates (Vandrey and Haney 2009). While partial agonists have been proposed to block the reinforcing effects of other drugs of abuse like opioids and nicotine (Koob and Mason 2016), no partial agonists have been found to reduce cannabis use.

Many different pharmacological treatments have been investigated for reduction of cannabis withdrawal symptoms, primarily through modulation of cannabinoid receptors but also through other neurotransmitter systems including glutamate, dopamine, norepinephrine, serotonin, and GABA (Balter et al. 2014; Levin et al. 2016; Brezing and Levin 2018). In their comprehensive review of the different pharmacological treatments for CUD and cannabis withdrawal, Brezing and Levin (2018) conclude that therapies targeting specific symptoms of withdrawal (such as anxiety, irritability, sleep disturbances, and decreased appetite) should be administered in conjunction with treatments that target reduction in cannabis use and prevention of relapse. Promising candidates for treatment of CUD that prevent relapse include naltrexone, gabapentin, and N-acetylcysteine (NAC) (Mason et al. 2012; Brezing and Levin 2018). The greatest reduction in multiple withdrawal symptoms has been shown with treatment using CB1R agonists such as dronabinol (oral THC), nabixmols (a combination of THC and CBD), and nabilone (Balter et al. 2014; Brezing and Levin 2018); surprisingly, previous studies have not shown cannabidiol as a potential treatment for cannabis withdrawal despite its anxiolytic effects (Brezing and Levin 2018). With CB1R agonists as potential treatments, it is necessary to consider the abuse potential of these drugs. Dronabinol, nabilone, and nabixmols seem to have a lower abuse potential than smoked cannabis (Allsop et al. 2015; Tsang and Giudice 2016), but in one study of cannabinoid replacement therapy, dronabinol and nabixmol had higher self-reports of liking than placebo drugs (Allsop et al. 2015).

NAC is being investigated as an anticraving agent in cannabis addiction therapy due to its regulatory role in glutamate and dopamine signaling (Samuni et al. 2013). NAC helps regulate the intra- and extracellular levels of glutamate through the cysteine-glutamate antiporter. Increased extracellular glutamate levels activate inhibitory metabotropic glutamate receptors, reducing glutamate neurotransmission (Samuni et al. 2013). The upregulation of glutamate signaling during the anticipation/preoccupation phase may be counteracted with NAC treatment, reducing clinical symptoms of craving and therefore reducing relapse rates. A 2014 review article summarizes two studies that evaluated NAC therapy in CUD. In one study, the placebo cohort reported twice as many positive urine cannabinoid tests as compared to the NAC cohort (Asevedo et al. 2014). The other study did not report group differences in positive urine tests, but did find a significant reduction in self-reported cannabis craving in the treatment group (Asevedo et al. 2014). These studies reinforce the role of glutamate upregulation during cannabis abstinence on clinical outcomes such as craving and relapse.

## Discussion

After examining the acute and long-term effects of cannabis, CUD appears to conform to the general patterns of changes described in the Koob and Volkow model of addiction. Previous preclinical and clinical studies indicate that features of the three stages of drug addiction described by Koob and Volkow are also present in cannabis addiction (Fig. 1), although these findings may not be as robust as other drugs of abuse.

As described in the Koob and Volkow model (2016), most drugs of abuse result in the hyperactivation of the mesocorticolimbic dopaminergic reward pathway in the binge-intoxication stage of addiction. This hyperactivation seems to be present in cannabis addiction but to a lower extent. Acute THC administration elicits striatal DA release in animals (Bloomfield et al. 2016) and THC challenges were shown to increase striatal DA transmission in humans (Stokes et al. 2010; Bossong et al. 2015); although other studies have found no THC-induced increases in striatal DA (Barkus et al. 2011; Urban et al. 2012). Additionally, there are no baseline differences in dopamine D2/D3 receptor availability between cannabis users and healthy controls (Volkow et al. 2014c; van de Giessen et al. 2017), a finding that does not parallel addiction to other drugs of abuse (including cocaine, alcohol, methamphetamine, nicotine, or heroin) which is associated with substantial reductions in D2R availability in the ventral striatum (Wang et al. 1997; Volkow et al. 2001, 2014c, 2017c; Martinez et al. 2012; Albrecht et al. 2013; Tomasi et al. 2015a; Wiers et al. 2016a; Ashok et al. 2017). Nonetheless, as with other drugs of abuse, chronic cannabis use still results in blunted dopamine reactivity to a stimulant challenge (Volkow et al. 2014c; van de Giessen et al. 2017).

This blunted stimulant-induced dopamine reactivity has been associated with negative emotionality (Volkow et al. 2014c) a key feature of withdrawal/negative affect stage described by Koob and Volkow (2016). With the addition of withdrawal as a symptom of CUD, it is evident that the development of cannabis addiction parallels addiction to other drugs of abuse. In addition, chronic cannabis use has been associated with affect dysregulation that may involve changes in amygdala functioning (Filbey et al. 2013; Heitzeg et al. 2015; Spechler et al. 2015). As with other drugs of abuse, cannabis seems to disrupt HPA axis function (Somaini et al. 2012; Cuttler et al. 2017), another key neuroadaptation of the withdrawal/negative affect stage described by Koob and Volkow (2016).

Chronic cannabis use is also associated with the presence of cannabis cue-induced craving after abstinence (Filbey et al. 2016; Norberg et al. 2016), a hallmark of the preoccupation/anticipation stage of the Koob and Volkow framework (2016). The presence of cannabis cue-induced craving seems to be related to the loss of executive control over excessive salience for cannabis (Norberg et al. 2016). In addition, chronic cannabis use has been linked to impaired memory and IQ, suggesting changes in executive functioning after chronic cannabis use. However, IQ deficits appear to be present before initiation of cannabis use which may suggest that lower IQ could be a risk factor for cannabis addiction (Jackson et al. 2016).

Interestingly, chronic cannabis use is associated with a downregulation of CB1R – THC’s target receptor – that is restored after 4 weeks of abstinence in humans (Hirvonen et al. 2012). This pattern of abstinence-induced changes in target receptor density is also seen after abstinence from other drugs of abuse such as heroin, stimulants, and alcohol (in humans and animals) but with some caveats: the changes found are not consistent across brain regions for every drug and abstinence periods are not congruent between studies (Wang et al. 2012; Seip-Cammack et al. 2013; Ashok et al. 2017; Volkow et al. 2017c). Future studies should examine to whether changes in target receptors after abstinence are comparable across brain regions and if they follow the same time course in CUD and other SUD.

Future studies should also investigate if there are other features of the addiction framework proposed by Koob and Volkow in cannabis addiction. Specifically, more longitudinal studies should investigate behavioral and mood changes (such as changes in IQ or the presence of a mood disorder) before and after the onset of cannabis use to determine whether variations in behavior and mood are risk factors or the result of cannabis addiction rather than a consequence. Additionally, with the increasing potency of THC in street cannabis (ElSohly et al. 2016), it is necessary to evaluate whether long-term changes may be related to the THC content of cannabis. Future studies should also investigate the specific neurocircuitry Koob and Volkow (2016) implicate in the three stages of addiction: specifically, how cannabis use impacts glutamate signaling in the VTA (disrupted during binge/intoxication) and PFC (disrupted during preoccupation/craving) and acetylcholine signaling in the habenula (disrupted during withdrawal/negative affect).

Future research should also consider whether THC’s effects on neurons and microglia are related to addiction. Previous research indicates that chronic THC exposure in animals seems to activate microglia and produce neuroinflammation that may underlie some of the cognitive deficits associated with CUD (Melis et al. 2017); additionally, changes in neuron and glia morphology after chronic cannabis exposure may also contribute to the persistent cognitive and behavioral deficits linked to CUD (Cutando et al. 2013; Kolb et al. 2018). Therefore, future studies should investigate whether chronic THC exposure in animals and humans is linked to changes in various cell types in the brain that contribute to cannabis addiction through neuroinflammation. THC has also been shown to have immunosuppressant properties in animals (Suarez-Pinilla et al. 2014) while cannabis use has been associated with adverse cardiovascular effects in humans (Pacher et al. 2017; Goyal et al. 2017; Thomas et al. 2018); these peripheral effects could be another line of future research.

Although further research is necessary (Box 1), the findings summarized here indicate that neurobiological changes in CUD seem to parallel those in other addictions, albeit to a lesser extent in some brain systems. This is critical in light of recent findings demonstrating an increase in cannabis use and CUD and a corresponding decrease in the perceived risk of cannabis (Carliner et al. 2017; Hasin 2018).

**Table:** 

**Box 1.** Questions for future research
• Do changes in CBIR density after abstinence from cannabis parallel changes in target receptors of other drugs of abuse?
• Are behavioral and mood variations associated with cannabis use a risk factor or consequence of cannabis addiction?
• Are long-term behavioral and neurophysiological changes related to the THC content in cannabis?
• Is cannabis use associated with long-term changes in glutamate signaling as seen in other drugs of abuse?
• Is cannabis use associated with disruptions in the amygdala and habenula as seen with other drugs of abuse?

## References

[CR1] Albrecht DS, Skosnik PD, Vollmer JM, Brumbaugh MS, Perry KM, Mock BH, Zheng QH, Federici LA, Patton EA, Herring CM, Yoder KK (2013). Striatal D2/D3 receptor availability is inversely correlated with cannabis consumption in chronic marijuana users. Drug Alcohol Depend.

[CR2] Allsop D, Lintzeris N, Copeland J, Dunlop A, McGregor I (2015). Cannabinoid replacement therapy (CRT): nabiximols (Sativex) as a novel treatment for cannabis withdrawal. Clin Pharmacol Ther.

[CR3] Asevedo E, Mendes AC, Berk M, Brietzke E (2014). Systematic review of N-acetylcysteine in the treatment of addictions. Rev Bras Psiquiatr.

[CR4] Ashok AH, Mizuno Y, Volkow ND, Howes OD (2017). Association of stimulant use with dopaminergic alterations in users of cocaine, amphetamine, or methamphetamine: a systematic review and meta-analysis. JAMA Psychiat.

[CR5] Balter RE, Cooper ZD, Haney M (2014). Novel pharmacologic approaches to treating cannabis use disorder. Curr Addict Rep.

[CR6] Barkus E, Morrison PD, Vuletic D, Dickson JC, Ell PJ, Pilowsky LS, Brenneisen R, Holt DW, Powell J, Kapur S, Murray RM (2011). Does intravenous Delta9-tetrahydrocannabinol increase dopamine release? A SPET study. J Psychopharmacol.

[CR7] Bayrakçi A, Sert E, Zorlu N (2015). Facial emotion recognition deficits in abstinent cannabis dependent patients. Compr Psychiatry.

[CR8] Becker MP, Collins PF, Luciana M (2014). Neurocognition in college-aged daily marijuana users. J Clin Exp Neuropsychol.

[CR9] Bhattacharyya S, Atakan Z, Martin-Santos R, A. Crippa J, K. McGuire P (2012). Neural mechanisms for the cannabinoid modulation of cognition and affect in man: a critical review of neuroimaging studies. Curr Pharm Des.

[CR10] Bloomfield MAP, Morgan CJA, Kapur S, Curran HV, Howes OD (2014). The link between dopamine function and apathy in cannabis users: an [ 18F]-DOPA PET imaging study. Psychopharmacology.

[CR11] Bloomfield MAP, Ashok AH, Volkow ND, Howes OD (2016). The effects of Δ9-tetrahydrocannabinol on the dopamine system. Nature.

[CR12] Bossong MG, Mehta MA, Van Berckel BNM (2015). Further human evidence for striatal dopamine release induced by administration of δ9-tetrahydrocannabinol (THC): selectivity to limbic striatum. Psychopharmacology.

[CR13] Brezing CA, Levin FR (2018). The current state of pharmacological treatments for cannabis use disorder and withdrawal. Neuropsychopharmacology.

[CR14] Broyd SJ, van Hell HH, Beale C, Yücel M, Solowij N (2016). Acute and chronic effects of cannabinoids on human cognition-a systematic review. Biol Psychiatry.

[CR15] Budney AJ, Vandrey RG, Hughes JR, Thostenson JD, Bursac Z (2008). Comparison of Cannabis and tobacco withdrawal: severity and contribution to relapse. J Subst Abus Treat.

[CR16] Caballero A, Tseng KY (2012). Association of cannabis use during adolescence, prefrontal CB1 receptor signaling, and schizophrenia. Front Pharmacol.

[CR17] Caprioli Daniele, Justinova Zuzana, Venniro Marco, Shaham Yavin (2018). Effect of Novel Allosteric Modulators of Metabotropic Glutamate Receptors on Drug Self-administration and Relapse: A Review of Preclinical Studies and Their Clinical Implications. Biological Psychiatry.

[CR18] Carliner Hannah, Brown Qiana L., Sarvet Aaron L., Hasin Deborah S. (2017). Cannabis use, attitudes, and legal status in the U.S.: A review. Preventive Medicine.

[CR19] Colizzi M, McGuire P, Pertwee RG, Bhattacharyya S (2016). Effect of cannabis on glutamate signalling in the brain: a systematic review of human and animal evidence. Neurosci Biobehav Rev.

[CR20] Crippa JAS, Derenusson GN, Chagas MHN, Atakan Z, Martín-Santos R, Zuardi AW, Hallak JEC (2012). Pharmacological interventions in the treatment of the acute effects of cannabis: a systematic review of literature. Harm Reduct J.

[CR21] Curran HV, Freeman TP, Mokrysz C, Lewis DA, Morgan CJA, Parsons LH (2016). Keep off the grass? Cannabis, cognition and addiction. Nat Rev Neurosci.

[CR22] Cutando L, Busquets-Garcia A, Puighermanal E, Gomis-González M, Delgado-García JM, Gruart A, Maldonado R, Ozaita A (2013). Microglial activation underlies cerebellar deficits produced by repeated cannabis exposure. J Clin Invest.

[CR23] Cuttler C, Spradlin A, Nusbaum AT, Whitney P, Hinson JM, McLaughlin RJ (2017). Blunted stress reactivity in chronic cannabis users. Psychopharmacology.

[CR24] D’Souza DC, Cortes-Briones JA, Ranganathan M, Thurnauer H, Creatura G, Surti T, Planeta B, Neumeister A, Pittman B, Normandin MD, Kapinos M, Ropchan J, Huang Y, Carson RE, Skosnik PD (2016). Rapid changes in cannabinoid 1 receptor availability in cannabis-dependent male subjects after abstinence from cannabis. Biol Psychiatry Cogn Neurosci Neuroimaging.

[CR25] Danovitch I, Gorelick DA (2012). State of the art treatments for cannabis dependence. Psychiatr Clin North Am.

[CR26] Davis JP, Smith DC, Morphew JW, Lei X, Zhang S (2016). Cannabis withdrawal, posttreatment abstinence, and days to first cannabis use among emerging adults in substance use treatment. J Drug Issues.

[CR27] Dorard G, Berthoz S, Phan O, Corcos M, Bungener C (2008). Affect dysregulation in cannabis abusers: a study in adolescents and young adults. Eur Child Adolesc Psychiatry.

[CR28] Dow-Edwards D, Silva L (2017). Endocannabinoids in brain plasticity: cortical maturation, HPA axis function and behavior. Brain Res.

[CR29] Draycott B, Loureiro M, Ahmad T, Tan H, Zunder J, Laviolette SR (2014). Cannabinoid transmission in the prefrontal cortex bi-phasically controls emotional memory formation via functional interactions with the ventral tegmental area. J Neurosci.

[CR30] ElSohly MA, Mehmedic Z, Foster S (2016). Changes in cannabis potency over the last 2 decades (1995–2014): analysis of current data in the United States. Biol Psychiatry.

[CR31] Filbey FM, Dunlop J, Myers US (2013). Neural effects of positive and negative incentives during marijuana withdrawal. PLoS One.

[CR32] Filbey FM, Dunlop J, Ketcherside A, Baine J, Rhinehardt T, Kuhn B, DeWitt S, Alvi T (2016). fMRI study of neural sensitization to hedonic stimuli in long-term, daily cannabis users. Hum Brain Mapp.

[CR33] Food and Drug Administration (2007) FDA briefing document. NDA 21-888. Zimulti (rimonabant) tablets, 20 mg. Sanofi Aventis. Advisory Committee–June 13, 2007

[CR34] Gates P, Albertella L, Copeland J (2016). Cannabis withdrawal and sleep: a systematic review of human studies. Subst Abus.

[CR35] Gilman JM, Kuster JK, Lee S, Lee MJ, Kim BW, Makris N, van der Kouwe A, Blood AJ, Breiter HC (2014). Cannabis use is quantitatively associated with nucleus accumbens and amygdala abnormalities in young adult recreational users. J Neurosci.

[CR36] Goyal H, Awad HH, Ghali JK (2017). Role of cannabis in cardiovascular disorders. J Thorac Dis.

[CR37] Hasin DS (2018). US epidemiology of cannabis use and associated problems. Neuropsychopharmacology.

[CR38] Hasin DS, Saha TD, Kerridge BT, Goldstein RB, Chou SP, Zhang H, Jung J, Pickering RP, Ruan WJ, Smith SM, Huang B, Grant BF (2015). Prevalence of marijuana use disorders in the United States between 2001–2002 and 2012–2013. JAMA Psychiat.

[CR39] Heitzeg MM, Cope LM, Martz ME, Hardee JE, Zucker RA (2015). Brain activation to negative stimuli mediates a relationship between adolescent marijuana use and later emotional functioning. Dev Cogn Neurosci.

[CR40] Henry KL, Augustyn MB (2017). Intergenerational continuity in cannabis use: the role of parent’s early onset and lifetime disorder on child’s early onset. J Adolesc Health.

[CR41] Hirvonen J, Goodwin RS, Li C-T, Terry GE, Zoghbi SS, Morse C, Pike VW, Volkow ND, Huestis MA, Innis RB (2012). Reversible and regionally selective downregulation of brain cannabinoid CB1 receptors in chronic daily cannabis smokers. Mol Psychiatry.

[CR42] Jackson NJ, Isen JD, Khoddam R, Irons D, Tuvblad C, Iacono WG, McGue M, Raine A, Baker LA (2016). Impact of adolescent marijuana use on intelligence: results from two longitudinal twin studies. Proc Natl Acad Sci U S A.

[CR43] Jasinska AJ, Stein EA, Kaiser J, Naumer MJ, Yalachkov Y (2014). Factors modulating neural reactivity to drug cues in addiction: a survey of human neuroimaging studies. Neurosci Biobehav Rev.

[CR44] Karila L, Roux P, Rolland B, Benyamina A, Reynaud M, Aubin HJ, Lancon C (2014). Acute and long-term effects of cannabis use: a review. Curr Pharm Des.

[CR45] Katsidoni V, Kastellakis A, Panagis G (2013). Biphasic effects of Δ9-tetrahydrocannabinol on brain stimulation reward and motor activity. Int J Neuropsychopharmacol.

[CR46] Katz G, Lobel T, Tetelbaum A, Raskin S (2014). Cannabis withdrawal - a new diagnostic category in DSM-5. Isr J Psychiatry Relat Sci.

[CR47] Koenders L, Cousijn J, Vingerhoets WAM, van den Brink W, Wiers RW, Meijer CJ, Machielsen MWJ, Veltman DJ, Goudriaan AE, de Haan L (2016). Grey matter changes associated with heavy cannabis use: a longitudinal sMRI study. PLoS One.

[CR48] Kolb Bryan, Li Yilin, Robinson Terry, Parker Linda A. (2017). THC alters alters morphology of neurons in medial prefrontal cortex, orbital prefrontal cortex, and nucleus accumbens and alters the ability of later experience to promote structural plasticity. Synapse.

[CR49] Koob GF, Mason BJ (2016). Existing and future drugs for the treatment of the dark side of addiction. Annu Rev Pharmacol Toxicol.

[CR50] Koob GF, Volkow ND (2016). Neurobiology of addiction: a neurocircuitry analysis. Lancet Psychiatry.

[CR51] Lefever TW, Marusich JA, Antonazzo KR, Wiley JL (2014). Evaluation of WIN 55,212-2 self-administration in rats as a potential cannabinoid abuse liability model. Pharmacol Biochem Behav.

[CR52] Levin FR, Mariani JJ, Pavlicova M, Brooks D, Glass A, Mahony A, Nunes EV, Bisaga A, Dakwar E, Carpenter KM, Sullivan MA, Choi JC (2016). Dronabinol and lofexidine for cannabis use disorder: a randomized, double-blind, placebo-controlled trial. Drug Alcohol Depend.

[CR53] Lorenzetti V, Solowij N, Whittle S, Fornito A, Lubman DI, Pantelis C, Yücel M (2015). Gross morphological brain changes with chronic, heavy cannabis use. Br J Psychiatry.

[CR54] Maldonado R, Rodriguez de Fonseca F (2002). Cannabinoid addiction: behavioral models and neural correlates. J Neurosci.

[CR55] Maldonado R, Berrendero F, Ozaita A, Robledo P (2011). Neurochemical basis of cannabis addiction. Neuroscience.

[CR56] Manza P, Tomasi D, Volkow ND (2018). Subcortical local functional hyperconnectivity in cannabis dependence. Biol Psychiatry Cogn Neurosci Neuroimaging.

[CR57] Martinez D, Saccone PA, Liu F, Slifstein M, Orlowska D, Grassetti A, Cook S, Broft A, van Heertum R, Comer SD (2012). Deficits in dopamine D 2 receptors and presynaptic dopamine in heroin dependence: commonalities and differences with other types of addiction. Biol Psychiatry.

[CR58] Martz ME, Trucco EM, Cope LM, Hardee JE, Jester JM, Zucker RA, Heitzeg MM (2016). Association of marijuana use with blunted nucleus accumbens response to reward anticipation. JAMA Psychiat.

[CR59] Mason BJ, Crean R, Goodell V, Light JM, Quello S, Shadan F, Buffkins K, Kyle M, Adusumalli M, Begovic A, Rao S (2012). A proof-of-concept randomized controlled study of gabapentin: effects on cannabis use, withdrawal and executive function deficits in cannabis-dependent adults. Neuropsychopharmacology.

[CR60] Meier MH, Caspi A, Danese A, Fisher HL, Houts R, Arseneault L, Moffitt TE (2018). Associations between adolescent cannabis use and neuropsychological decline: a longitudinal co-twin control study. Addiction.

[CR61] Melis M, Frau R, Kalivas PW, Spencer S, Chioma V, Zamberletti E, Rubino T, Parolaro D (2017). New vistas on cannabis use disorder. Neuropharmacology.

[CR62] Moreno-Lopez L, Stamatakis EA, Fernandez-Serrano MJ (2012). Neural correlates of the severity of cocaine, heroin, alcohol, MDMA and cannabis use in polysubstance abusers: a resting-PET brain metabolism study. PLoS One.

[CR63] Morgan CJ, Freeman TP, Schafer GL, Curran HV (2010). Cannabidiol attenuates the appetitive effects of Δ9-tetrahydrocannabinol in humans smoking their chosen cannabis. Neuropsychopharmacology.

[CR64] Ng Cheong Ton JM, Gerhardt GA, Friedemann M, Etgen AM, Rose GM, Sharpless NS, Gardner EL (1988). The effects of delta 9-tetrahydrocannabinol on potassium-evoked release of dopamine in the rat caudate nucleus: an in vivo electrochemical and in vivo microdialysis study. Brain Res.

[CR65] Norberg MM, Kavanagh DJ, Olivier J, Lyras S (2016). Craving cannabis: a meta-analysis of self-report and psychophysiological cue-reactivity studies. Addiction.

[CR66] Oleson EB, Cheer JF (2012). A brain on cannabinoids: the role of dopamine release in reward seeking. Cold Spring Harb Perspect Med.

[CR67] Pacher P, Steffens S, Haskó G, Schindler TH, Kunos G (2017). Cardiovascular effects of marijuana and synthetic cannabinoids: the good, the bad, and the ugly. Nat Rev Cardiol.

[CR68] Panlilio L, Goldberg S, Justinova Z (2015). Cannabinoid abuse and addiction: clinical and preclinical findings. Clin Pharmacol Ther.

[CR69] Parsons LH, Hurd YL (2015). Endocannabinoid signalling in reward and addiction. Nat Rev Neurosci.

[CR70] Paule MG, Allen RR, Bailey JR, Scallet AC, Ali SF, Brown RM, Slikker W Jr (1992). Chronic marijuana smoke exposure in the rhesus monkey. II: effects on progressive ratio and conditioned position responding. J Pharmacol Exp Ther.

[CR71] Pujol J, Blanco-Hinojo L, Batalla A, López-Solà M, Harrison BJ, Soriano-Mas C, Crippa JA, Fagundo AB, Deus J, de la Torre R, Nogué S, Farré M, Torrens M, Martín-Santos R (2014). Functional connectivity alterations in brain networks relevant to self-awareness in chronic cannabis users. J Psychiatr Res.

[CR72] Renard J, Vitalis T, Rame M, Krebs MO, Lenkei Z, le Pen G, Jay TM (2016). Chronic cannabinoid exposure during adolescence leads to long-term structural and functional changes in the prefrontal cortex. Eur Neuropsychopharmacol.

[CR73] Rodriguez de Fonseca F, Carrera MR, Navarro M (1997). Activation of corticotropin-releasing factor in the limbic system during cannabinoid withdrawal. Science.

[CR74] Samuni Y, Goldstein S, Dean OM, Berk M (2013). The chemistry and biological activities of N-acetylcysteine. Biochim Biophys Acta Gen Subj.

[CR75] Scherma M, Dessi C, Muntoni AL (2016). Adolescent Delta(9)-Tetrahydrocannabinol exposure alters WIN55,212-2 self-administration in adult rats. Neuropsychopharmacology.

[CR76] Seip-Cammack KM, Reed B, Zhang Y, Ho A, Kreek MJ (2013). Tolerance and sensitization to chronic escalating dose heroin following extended withdrawal in fischer rats: possible role of mu-opioid receptors. Psychopharmacology.

[CR77] Sevy S, Smith GS, Ma Y, Dhawan V, Chaly T, Kingsley PB, Kumra S, Abdelmessih S, Eidelberg D (2008). Cerebral glucose metabolism and D2/D3 receptor availability in young adults with cannabis dependence measured with positron emission tomography. Psychopharmacology.

[CR78] Somaini L, Manfredini M, Amore M, Zaimovic A, Raggi MA, Leonardi C, Gerra ML, Donnini C, Gerra G (2012). Psychobiological responses to unpleasant emotions in cannabis users. Eur Arch Psychiatry Clin Neurosci.

[CR79] Spechler PA, Orr CA, Chaarani B, Kan KJ, Mackey S, Morton A, Snowe MP, Hudson KE, Althoff RR, Higgins ST, Cattrell A, Flor H, Nees F, Banaschewski T, Bokde ALW, Whelan R, Büchel C, Bromberg U, Conrod P, Frouin V, Papadopoulos D, Gallinat J, Heinz A, Walter H, Ittermann B, Gowland P, Paus T, Poustka L, Martinot JL, Artiges E, Smolka MN, Schumann G, Garavan H, IMAGEN Consortium (2015). Cannabis use in early adolescence: evidence of amygdala hypersensitivity to signals of threat. Dev Cogn Neurosci.

[CR80] Stephens MAC, Wand G (2012). Stress and the HPA axis: role of glucocorticoids in alcohol dependence. Alcohol Res.

[CR81] Stokes PRA, Egerton A, Watson B, Reid A, Breen G, Lingford-Hughes A, Nutt DJ, Mehta MA (2010). Significant decreases in frontal and temporal [11C]-raclopride binding after THC challenge. NeuroImage.

[CR82] Suarez-Pinilla P, Lopez-Gil J, Crespo-Facorro B (2014). Immune system: a possible nexus between cannabinoids and psychosis. Brain Behav Immun.

[CR83] Tanda G, Goldberg SR (2003). Cannabinoids: reward, dependence, and underlying neurochemical mechanisms - a review of recent preclinical data. Psychopharmacology.

[CR84] Taylor D (2009). Withdrawal of Rimonabant--walking the tightrope of 21st century pharmaceutical regulation?. Curr Drug Saf.

[CR85] Thomas G, Kloner RA, Rezkalla S (2018). Adverse cardiovascular, cerebrovascular, and peripheral vascular effects of marijuana inhalation: what cardiologists need to know. Am J Cardiol.

[CR86] Tomasi D, Wang G-J, Wang R, Caparelli EC, Logan J, Volkow ND (2015). Overlapping patterns of brain activation to food and cocaine cues in cocaine abusers: association to striatal D2/D3 receptors. Hum Brain Mapp.

[CR87] Tomasi D, Wang GJ, Volkow ND (2015). Balanced modulation of striatal activation from D2/D3receptors in caudate and ventral striatum: disruption in cannabis abusers. Hum Brain Mapp.

[CR88] Tsang CC, Giudice MG (2016). Nabilone for the management of pain. Pharmacotherapy.

[CR89] Urban NBL, Slifstein M, Thompson JL, Xu X, Girgis RR, Raheja S, Haney M, Abi-Dargham A (2012). Dopamine release in chronic cannabis users: a [ 11C]raclopride positron emission tomography study. Biol Psychiatry.

[CR90] van de Giessen E, Weinstein JJ, Cassidy CM, Haney M, Dong Z, Ghazzaoui R, Ojeil N, Kegeles LS, Xu X, Vadhan NP, Volkow ND, Slifstein M, Abi-Dargham A (2017). Deficits in striatal dopamine release in cannabis dependence. Mol Psychiatry.

[CR91] Vandrey R, Haney M (2009). Pharmacotherapy for cannabis dependence: how close are we?. CNS Drugs.

[CR92] Volkow ND, Gillespie H, Mullani N, Tancredi L, Grant C, Valentine A, Hollister L (1996). Brain glucose metabolism in chronic marijuana users at baseline and during marijuana intoxication. Psychiatry Res.

[CR93] Volkow ND, Wang GJ, Fowler JS, Logan J, Hitzemann R, Ding YS, Pappas N, Shea C, Piscani K (1996). Decreases in dopamine receptors but not in dopamine transporters in alcoholics. Alcohol Clin Exp Res.

[CR94] Volkow ND, Wang GJ, Fowler JS, Logan J, Gatley SJ, Wong C, Hitzemann R, Pappas NR (1999). Reinforcing effects of psychostimulants in humans are associated with increases in brain dopamine and occupancy of D(2) receptors. J Pharmacol Exp Ther.

[CR95] Volkow ND, Wang GJ, Fowler JS, Hitzemann R, Angrist B, Gatley SJ, Logan J, Ding YS, Pappas N (1999). Association of methylphenidate-induced craving with changes in right striato-orbitofrontal metabolism in cocaine abusers: implications in addiction. Am J Psychiatry.

[CR96] Volkow ND, Chang L, Wang GJ, Fowler JS, Ding YS, Sedler M, Logan J, Franceschi D, Gatley J, Hitzemann R, Gifford A, Wong C, Pappas N (2001). Low level of brain dopamine D2 receptors in methamphetamine abusers: association with metabolism in the orbitofrontal cortex. Am J Psychiatry.

[CR97] Volkow ND, Wang G-J, Maynard L, Fowler JS, Jayne B, Telang F, Logan J, Ding YS, Gatley SJ, Hitzemann R, Wong C, Pappas N (2002). Effects of alcohol detoxification on dopamine D2 receptors in alcoholics: a preliminary study. Psychiatry Res.

[CR98] Volkow ND, Wang G-J, Ma Y, Fowler JS, Wong C, Ding YS, Hitzemann R, Swanson JM, Kalivas P (2005). Activation of orbital and medial prefrontal cortex by methylphenidate in cocaine-addicted subjects but not in controls: relevance to addiction. J Neurosci.

[CR99] Volkow ND, Baler RD, Compton WM, Weiss SRB (2014). Adverse health effects of marijuana use. N Engl J Med.

[CR100] Volkow ND, Tomasi D, Wang G-J, Logan J, Alexoff DL, Jayne M, Fowler JS, Wong C, Yin P, du C (2014). Stimulant-induced dopamine increases are markedly blunted in active cocaine abusers. Mol Psychiatry.

[CR101] Volkow ND, Wang G-J, Telang F, Fowler JS, Alexoff D, Logan J, Jayne M, Wong C, Tomasi D (2014). Decreased dopamine brain reactivity in marijuana abusers is associated with negative emotionality and addiction severity. Proc Natl Acad Sci.

[CR102] Volkow ND, Swanson JM, Evins AE, DeLisi LE, Meier MH, Gonzalez R, Bloomfield MAP, Curran HV, Baler R (2016). Effects of cannabis use on human behavior, including cognition, motivation, and psychosis: a review. JAMA Psychiat.

[CR103] Volkow ND, Hampson AJ, Baler RD (2017). Don’t worry, be happy: endocannabinoids and cannabis at the intersection of stress and reward. Annu Rev Pharmacol Toxicol.

[CR104] Volkow Nora D., Koob George F., Croyle Robert T., Bianchi Diana W., Gordon Joshua A., Koroshetz Walter J., Pérez-Stable Eliseo J., Riley William T., Bloch Michele H., Conway Kevin, Deeds Bethany G., Dowling Gayathri J., Grant Steven, Howlett Katia D., Matochik John A., Morgan Glen D., Murray Margaret M., Noronha Antonio, Spong Catherine Y., Wargo Eric M., Warren Kenneth R., Weiss Susan R.B. (2018). The conception of the ABCD study: From substance use to a broad NIH collaboration. Developmental Cognitive Neuroscience.

[CR105] Volkow ND, Wiers CE, Shokri-Kojori E, Tomasi D, Wang GJ, Baler R (2017). Neurochemical and metabolic effects of acute and chronic alcohol in the human brain: studies with positron emission tomography. Neuropharmacology.

[CR106] Wang GJ, Volkow ND, Fowler JS, Logan J, Abumrad NN, Hitzemann RJ, Pappas NS, Pascani K (1997). Dopamine D2 receptor availability in opiate-dependent subjects before and after naloxone-precipitated withdrawal. Neuropsychopharmacology.

[CR107] Wang GJ, Smith L, Volkow ND, Telang F, Logan J, Tomasi D, Wong CT, Hoffman W, Jayne M, Alia-Klein N, Thanos P, Fowler JS (2012). Decreased dopamine activity predicts relapse in methamphetamine abusers. Mol Psychiatry.

[CR108] Weiland BJ, Thayer RE, Depue BE, Sabbineni A, Bryan AD, Hutchison KE (2015). Daily marijuana use is not associated with brain morphometric measures in adolescents or adults. J Neurosci.

[CR109] Wiers CE, Cabrera E, Skarda E (2016). PET imaging for addiction medicine: from neural mechanisms to clinical considerations. Prog Brain Res.

[CR110] Wiers CE, Shokri-Kojori E, Wong CT, Abi-Dargham A, Demiral ŞB, Tomasi D, Wang GJ, Volkow ND (2016). Cannabis abusers show hypofrontality and blunted brain responses to a stimulant challenge in females but not in males. Neuropsychopharmacology.

[CR111] Wiers CE, Cabrera EA, Tomasi D, Wong CT, Demiral ŞB, Kim SW, Wang GJ, Volkow ND (2017). Striatal dopamine D2/D3 receptor availability varies across smoking status. Neuropsychopharmacology.

[CR112] Wijayendran SB, O’Neill A, Bhattacharyya S (2016). The effects of cannabis use on salience attribution: a systematic review. Acta Neuropsychiatr.

[CR113] Wilson SJ, Sayette MA (2015). Neuroimaging craving: urge intensity matters. Addiction.

[CR114] Yip SW, DeVito EE, Kober H (2014). Pretreatment measures of brain structure and reward-processing brain function in cannabis dependence: an exploratory study of relationships with abstinence during behavioral treatment1. Drug Alcohol Depend.

[CR115] Zanda MT, Fattore L (2018). Old and new synthetic cannabinoids: lessons from animal models. Drug Metab Rev.

[CR116] Zimmermann K, Yao S, Heinz M, Zhou F, Dau W, Banger M, Weber B, Hurlemann R, Becker B (2018). Altered orbitofrontal activity and dorsal striatal connectivity during emotion processing in dependent marijuana users after 28 days of abstinence. Psychopharmacology.

